# Effectiveness of clindamycin-based exposure strategies in experimental mature staphylococcal biofilms

**DOI:** 10.1128/spectrum.01947-24

**Published:** 2025-01-30

**Authors:** S. C. J. van Dun, M. Verheul, B. G. C. W. Pijls, H. Scheper, A. M. van der Does, P. H. Nibbering, M. G. J. de Boer

**Affiliations:** 1 Center for Infectious Diseases, Lab of Infectious Diseases, Leiden University Medical Center, Leiden, the Netherlands; 2 Department of Orthopaedics, Leiden University Medical Center, Leiden, the Netherlands; 3 PulmoScience Lab, Department of Pulmonology, Leiden University Medical Center, Leiden, the Netherlands; The Hebrew University of Jerusalem, Rehovot, Israel

**Keywords:** biofilm, PJI, *Staphylococcus aureus*, clindamycin, rifampicin

## Abstract

Due to increasing antimicrobial resistance and side effects caused by current standard antimicrobial regimens used for treatment of prosthetic joint infection (PJI), alternative options are urgently needed. We aimed to investigate the effect of clindamycin in different exposure strategies against *Staphylococcus aureus* in an *in vitro* mature biofilm model. In short, 7-day biofilms were generated on polystyrene plates and titanium–aluminum–vanadium discs using a clinical *S. aureus* PJI isolate. Next, biofilms were exposed to clindamycin according to four strategies: single 24-h exposure; prolonged 48- or 72-h exposure; repeated 24-h exposures during a 4-day period, and sequential exposures of initial 24-h rifampicin-based therapy followed by 24-h exposure to clindamycin. The remaining bacterial load (colony-forming unit [CFU]/mL) after antibiotic exposure was assessed. Confocal laser scanning and atomic force microscopy were applied to evaluate the biofilm structure. Single exposure to clindamycin for 24 h or prolonged up to 72 h did not result in any relevant reduction in bacterial load. Repeated 24-h exposures demonstrated relevant reductions of >3 log CFU/mL at clindamycin concentrations ≥16 mg/L for the 3rd and 4th consecutive doses. Sequential rifampicin–ciprofloxacin combination exposure followed by clindamycin showed bacterial load reductions of 3- to 4-log CFU/mL, similar to continued rifampicin–ciprofloxacin exposure. This was achieved for concentrations equivalent to levels achieved after standard dosing of clindamycin in clinical practice. These experimental findings support that clindamycin monotherapy is not an optimal choice when starting antimicrobial treatment of PJI but that later on, rifampicin may be safely switched to clindamycin in patients with PJI who do not tolerate prolonged rifampicin-based treatment.

**IMPORTANCE:**

Rifampicin—in combination with another antibiotics—is recommended in all guidelines as first choice treatment of prosthetic joint infections (PJIs), despite adverse interactions and side effects associated with this antibiotic. In a search for alternative approaches, the switch to clindamycin in patients after rifampicin-based antibiotic treatment was found to be effective in some recent observational clinical studies. In our in vitro study, we determined the effect of clindamycin on *Staphylococcus aureus* in mature biofilms, to obtain further insight. Our study showed that clindamycin was effective in reducing mature biofilm-residing *S. aureus* after initial exposure to rifampicin–ciprofloxacin, while it was not effective as first treatment. These in vitro findings provide evidence for the hypothesis that rifampicin–ciprofloxacin can be successfully switched to clindamycin monotherapy in PJI patients in a later phase of treatment.

## INTRODUCTION

Prosthetic joint infections (PJIs) are a serious complication of orthopedic implant surgery, originating from microbial contamination during surgery or hematogenous seeding. These infections are often caused by *Staphylococcus aureus* or other staphylococcal species (1). A common characteristic of these microorganisms is the formation of biofilms on the implant surface. Biofilms are multicellular bacterial communities in a self-produced extracellular matrix. Bacteria within a biofilm are more protected against environmental stressors, such as antibiotics and host immune cells. The presence of biofilms in PJIs makes these infections difficult to treat, with treatment failure rates reaching 10%–50%, depending on the clinical situation (2). Rifampicin (RIF) is widely considered a highly effective, biofilm-penetrating antibiotic and is therefore recommended by nearly all guidelines as a first-choice regimen in treating PJIs. The main reasons to discontinue RIF-based treatment are the need for administration of comedication due to rapid resistance development in RIF monotherapy (3), drug–drug interactions between RIF and provided comedication, and potentially severe side effects such as renal problems and drug-induced hepatitis (4). The overall RIF resistance rate of *S. aureus* isolates remains low (0.6% across Europe [5], with exceptions for some countries). However, in PJIs, the incidence is higher (6), reaching 3% for *S. aureus*. Despite these disadvantages, the clinical approach has remained relatively similar over the last 20 years. In recent years, more research has focused on redesigning current antimicrobial treatment or finding new approaches to improve failure rates. When RIF-based treatment is not possible, alternative treatment strategies combining levofloxacin with clindamycin, minocycline, or co-trimoxazole have been used (7, 8). One currently debated strategy to improve the tolerability of antimicrobial treatment for patients is a switch to clindamycin monotherapy. Thus far, limited clinical data suggested that switching to clindamycin later in the course of antimicrobial treatment could be a promising approach. In one observational study, clindamycin-based treatment was as effective as other therapeutic strategies (9). Clindamycin has also been shown to be effective in treating other types of bone and joint infections, for example, spondylodiscitis and septic arthritis caused by staphylococcal species (10–12). However, its effectiveness and biological plausibility in the antimicrobial management of PJIs have yet to be established. The use of clindamycin in treatment of staphylococcal PJIs is currently under investigation in an ongoing multicenter randomized controlled clinical trial (13). There is a need for clinically relevant *in vitro* biofilm studies to analyze the effectiveness and optimal strategies of clindamycin-based treatment to support the clinical cases and observational studies. Therefore, this study aimed to investigate the effect of clindamycin in various exposure strategies and compare this to RIF-based treatment against *S. aureus* in an *in vitro* mature biofilm model.

## MATERIALS AND METHODS

### Bacterial culture

Clinical methicillin-susceptible *S. aureus* (MSSA) isolate LUH15392 from a PJI patient was stored in 20% glycerol at −80°C until use. Prior to experiments, stocks were thawed and spread on trypticase soy agar with 5% sheep blood plates (Biomerieux, Marcy-l’Étoile, France, 43009) and cultured overnight at 37°C. Thereafter, bacteria were cultured at 37°C to mid-log phase in tryptic soy broth (Oxoid, Basingstoke, Hampshire, UK, CM0129) for 2.5 h at 200 rpm. Bacteria were concentrated by centrifugation at 1,000 × g for 10 min and washed with Dulbecco’s phosphate-buffered saline (PBS, pH 7.4) (Sigma-Aldrich, Amsterdam, Netherlands, D8537). Ultimately, the bacteria were resuspended in brain heart infusion (BHI) broth (Oxoid, Basingstoke, Hampshire, UK, CM1135B) to the required concentrations based on the optical density at 600 nm, measured using Ultrospec 10 (GE Healthcare Bio-Sciences AB, Cambridge, UK).

### Antibiotics

RIF (R3501, Sigma-Aldrich, 822.94 g/mol) was diluted in DMSO (4 g/L) and ciprofloxacin (CIP) (PHR 1044, Sigma-Aldrich, 385.82 g/mol) in Milli-Q water (25.6 g/L), before storage at −20°C until use. Clindamycin (RVG 123224, Added Pharma, The Netherlands, 299–339 mosmol/kg) aliquots of 12-g/L stock were made and stored at room temperature until use. Antibiotic dilutions were made using PBS with 2% Tryptic Soy Broth (TSB).

### Killing assay of planktonic *S. aureus*

Bacterial strain LUH15392 was cultured as mentioned above and diluted in BHI to obtain a suspension of mid-log phase bacteria (1 × 10^7^ CFU/mL). Of this suspension, a volume of 100 µL was added to round-bottom polystyrene 96-well plates (Greiner Bio-One, Frickenhausen, Germany). Additionally, a twofold dilution series was prepared of the antibiotics CIP (0–102.4 mg/L), RIF (0–5 mg/L), and clindamycin (0–128 mg/L). Of these antibiotics, a volume of 100 µL was added to the wells with bacterial suspensions. Controls received BHI with the diluent of the antibiotics. Medium and diluent controls without bacteria were included to monitor possible contamination. Plates were sealed with non-breathable plastic film sealers (Amplistar, Westburg, Germany) and incubated for 24 h at 37°C and 200 rpm. Thereafter, well contents were resuspended to collect all bacteria. Bacterial suspensions were transferred to new round-bottom polystyrene 96-well plates and 10× dilution series were made in PBS. Bacterial suspensions of all dilutions were plated on Muller–Hinton agar plates (Oxoid, Basingstoke, Hampshire, UK, CM0337) in duplicate. After the spots were dried, plates were incubated overnight at 37°C. Thereafter, the number of CFU was counted. From this, the minimal bactericidal concentration (MBC) for RIF, CIP, and clindamycin was determined, which was defined as the lowest concentration needed to kill 99.9% of planktonic bacteria compared to the starting inoculum.

### Bacterial growth in the continuous presence of clindamycin

Bacterial suspensions at 1 × 10^7^ and 1 × 10^4^ CFU/mL were prepared in 10-mL BHI and incubated with clindamycin at concentrations ranging from 4 to 128 mg/L (vol/vol) at 37°C and 200 rpm. Samples of 100 µL were taken every hour up to 7 h and then at 24, 48, and 72 h. These samples were diluted and plated as described above. Thereafter, colony growth was assessed, and CFUs were counted.

### Anti-biofilm assays

**Fig 1:**
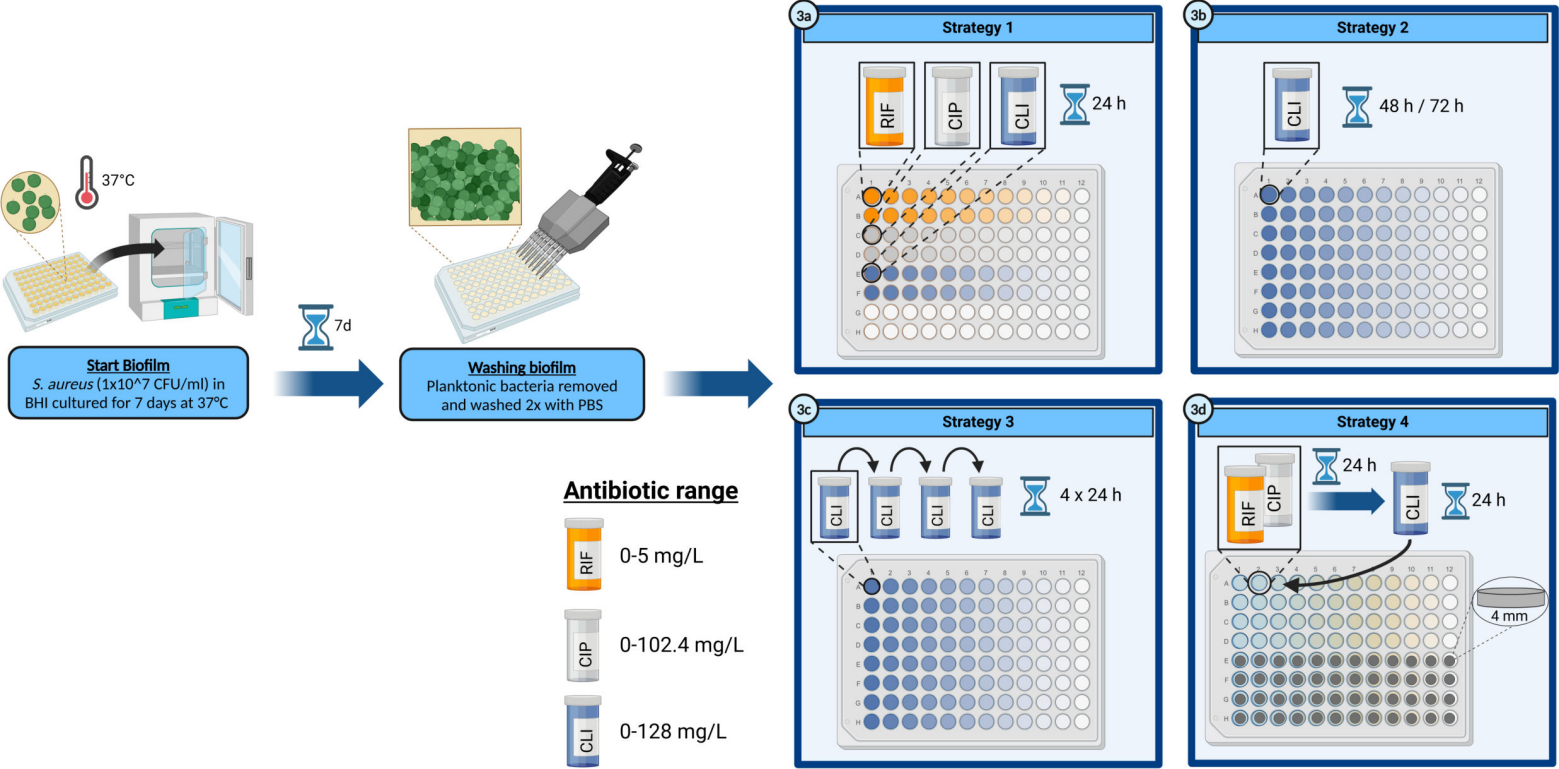
Experimental approach of the different anti-biofilm assays, with each number indicating a step in the procedure. The different treatment strategies are shown in step 3. Antibiotic concentration ranges (mg/L) are represented by color gradients and applied in twofold dilution series. CIP: ciprofloxacin; CLI: clindamycin; RIF: rifampicin; TAV: Ti-6Al-4V implant material discs (created with BioRender.com).

Bacteria were diluted in BHI to obtain a suspension of mid-log phase bacteria (1 × 10^7^ CFU/mL). A volume of 100 µL of this suspension was added to each well of a polystyrene flat-bottom 96-well microplate. Next, the plates were sealed with breathable rayon film sealers (VWR European, Leuven, Belgium) and incubated in a humidified environment for 7 days at 37°C to generate mature biofilms. For the treatment of these mature biofilms, four different strategies were assessed. A visual representation of the experimental approach of these different strategies is shown in Fig. 1. Strategy 1 is single 24-h antibiotic exposure. Strategy 2 builds on this by prolonging the exposure time to 48 or 72 h. This strategy was included, because clindamycin is a bacteriostatic agent, so it could take more time before any antibacterial effect is observed. Strategy three is a more clinically applicable concept, with repeated 24-h exposures over a 4-day period, as patients also receive multiple doses with specific intervals between administration. Strategy 4, the sequential treatment approach, combines the RIF-based treatment with a second phase switch to monotherapy with clindamycin. The various exposure strategies are summarized in Table S1.

#### Strategy 1: single antibiotic exposure

After 7-day incubation, wells were gently washed twice with 100 µL of PBS to remove the non-adherent bacterial cells from the wells. Remaining biofilms were subsequently exposed to various antibiotic concentrations in PBS + 2% TSB. Plates were sealed again and incubated for 24 h at 37°C. Biofilms exposed to medium without antibiotics were included for comparison. Medium and diluent controls without bacteria were included to monitor possible contamination. After exposure, culture medium was removed, and biofilms were gently washed twice with 100 µL of PBS. After washing, 100 µL of PBS were added to the wells. Plates were sealed with aluminium stickers and biofilms sonicated for 10 min at 40 kHz in Bransonic M Mechanical Bath 1800 (Branson Ultrasonics, Brookfield CT, USA) to disrupt the biofilms. Thereafter, well contents were resuspended to collect the previously biofilm-residing bacteria. Diluting and plating were done as mentioned before. Thereafter, the number of viable bacteria was assessed microbiologically and the results expressed as CFU/mL.

#### Strategy 2: prolonged antibiotic exposure

In this assay, the single exposure approach described in strategy 1 (2.5.1) was used, with the adaptation of exposure durations. The single 24-h exposure was changed to 48 or 72 h to assess the effect of clindamycin over a prolonged period of time.

#### Strategy 3: repeated antibiotic exposure

In this assay, the single-exposure approach described in strategy one was used, with the adaptation of treatment dosing. The single 24-h exposure was changed to two-, three- or 4-time 24 h to assess the effect of repeated exposure to clindamycin.

#### Strategy 4: sequential antibiotic exposure

In this assay, the single-exposure approach described in strategy one was used, with the adaptation of different antibiotics being applied in the order in which they would be administered in a clinical PJI treatment. Here, the assay was started with a 24-h exposure of mature biofilms to RIF/CIP combination. After this first treatment, liquid content was removed, and wells were gently washed twice with 100 µL of PBS. Next, 100 µL of clindamycin in various concentrations was added to the mature biofilms for 24 h.

In an extra set of experiments, this exposure strategy was performed with biofilms grown on sterile medical grade 5 titanium (6%), aluminium (4%), and vanadium (TAV; ISO 5832/3, Braun) discs (diameter 4 mm, height 1.5 mm). All discs were made specifically to fit in 96-well plates and are a standard component of the mature biofilm model, as described previously (14).

### Imaging of mature *S. aureus* biofilms after exposure to antibiotics

#### Confocal laser scanning microscopy

Viable- and non-viable bacteria within mature LUH15392 biofilms on µ-slide 8-well glass bottom chambered coverslip (80827, Ibidi) were visualized using LIVE/DEAD staining, that is, SYTO-9 green (5 mM, S34854, Invitrogen) and TO-PRO-3-iodide (1 mM, T3605, Invitrogen) red fluorescent nucleic acid-binding dyes, respectively (Fig. S1). Biofilms were washed twice with 300 µL of 0.9% NaCl, further referred to as saline, before incubating with 300 µL of SYTO-9 at 3 µM in saline for 30 min at RT in the dark. Thereafter, biofilms were washed once with 300 µL of saline and counterstained with 300 µL of TO-PRO-3-iodide 4 µM in saline for 15 min at RT in the dark. Again, biofilms were washed once with 300 µL of saline. SYTO-9 and TO-PRO-3-iodide stained biofilms were visualized in saline using 488 nm Ar^+^ laser line (emission collected at 500–590 nm) and the 633 nm He-Ne laser line (emission collected at 645–795 nm), respectively, using Andor Dragonfly 500 (Oxford instruments, Abingdon, UK). Vertical representation of biofilms was made with z-stacks (63× magnification), and images were analyzed with Imaris Software.

#### Atomic force microscopy

Mature LUH15392 biofilms formed on implant materials were fixed with 0.1% (vol/vol; in Milli-Q) glutaraldehyde for 4 h at room temperature, and samples were left to dry overnight after removing the fixative. Atomic force microscopy (AFM) images of the biofilm surface were acquired using a JPK NanoWizard IV (Berlin, Germany), according to a previously described protocol (15). Images were made at random locations on the implant discs (Fig. S1). The captured images were processed using JPKSPM Data Processing software version 6.1.191 (JPK BioAFM, Bruker Nano GmbH, Berlin, Germany).

### Statistical analysis

Data with a non-normal distribution, such as CFU counts, were log_10_ transformed to obtain a normal distribution (16). One- or two-way analysis of variance (ANOVA) statistical tests were performed to assess statistical differences between findings. All calculations and statistical tests were performed using GraphPad Prism version 9.3.1 (GraphPad Software, San Diego, CA, US).

## RESULTS

### Effect of PJI treatment-related standard and alternative antibiotics on planktonic *S. aureus*

**Fig 2:**
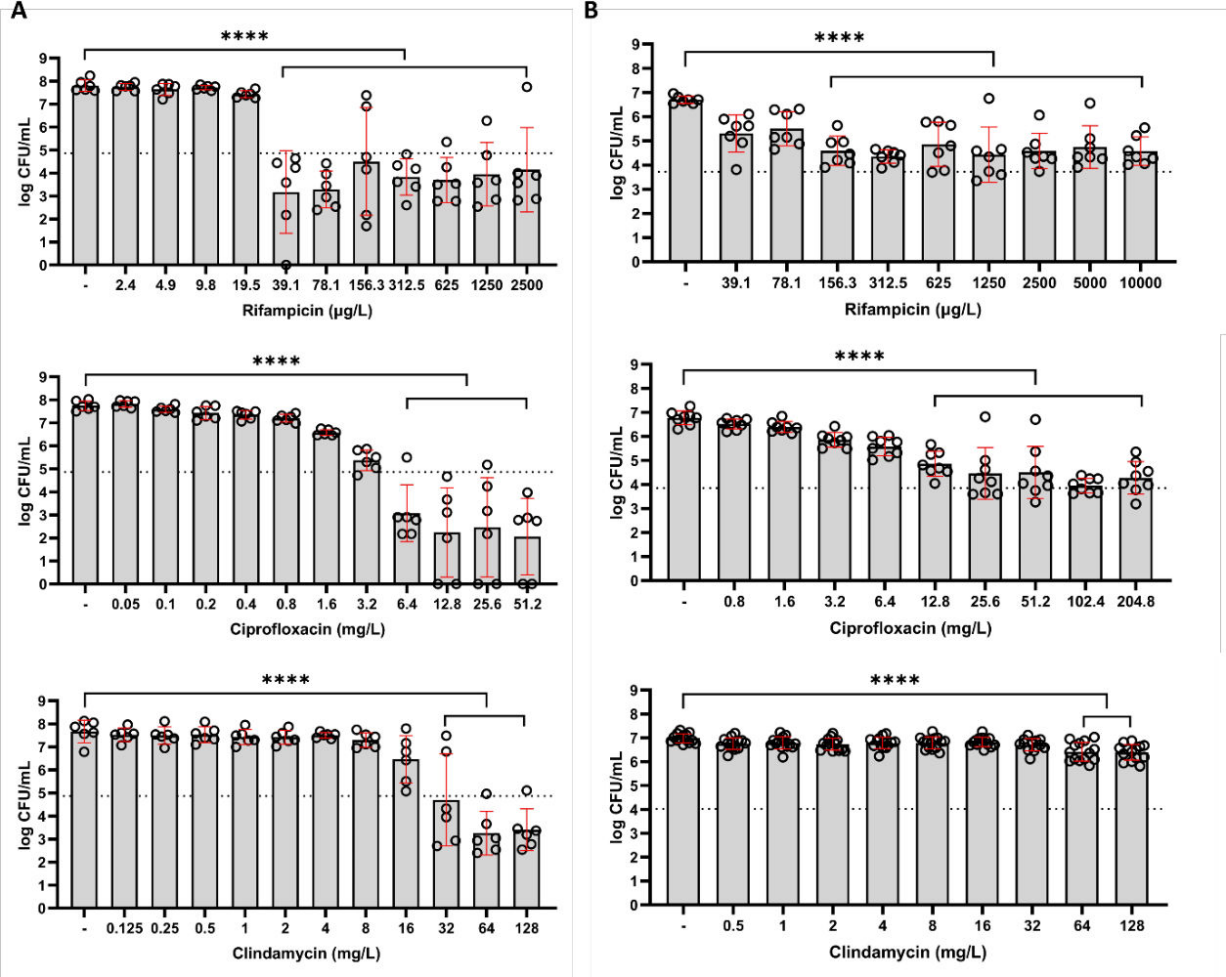
Bacterial load (log CFU/mL) after 24-h exposure to RIF (µg/l); CIP (mg/L), or clindamycin (mg/L) of (A) planktonic *S. aureus* LUH15392 and (B) mature 7-day biofilm-residing *S. aureus* LUH15392. Results are shown as individual values (*N* = 3 independent experiments, each performed in duplicate), bars indicate means, and error bars indicate standard deviation. The dotted line represents the 99.9% eradication threshold. One-way ANOVA was used to examine statistical differences between the control and antibiotic concentrations. ****P < 0.0001. CFU: colony-forming units.

To assess the antimicrobial effect of different PJI-related antibiotics, we exposed planktonic *S. aureus* LUH15392 to CIP (mg/L), RIF (µg/L), and clindamycin (mg/L) in a range of concentrations (Fig. 2A). This resulted in MBCs of 39.1-µg/L RIF and 6.4-mg/L CIP, respectively. Exposure to clindamycin resulted in an MBC of 32 mg/L. Furthermore, since clindamycin is a bacteriostatic antibiotic, we also assessed if clindamycin could inhibit planktonic bacterial growth for a prolonged period of time. This showed the capacity of clindamycin to inhibit bacterial growth at the MBC. It was even capable of growth inhibition at concentrations reachable in patients (4 and 8 mg/L), albeit for a shorter period of time (5 and 7 h, respectively) (Fig. S2A). However, bacterial counts did not reach a lower value than mean 5-log CFU/mL.

### Strategy 1: single antibiotic exposure

After assessing antibiotic efficacy against planktonic bacteria, we next determined the effectivity of these antibiotics against *S. aureus* LUH15392 in mature, 7-day biofilms (Fig. 2B). Results revealed that exposure to CIP, RIF, and clindamycin was less effective in reducing mature biofilm-residing *S. aureus* LUH15392 as compared to the planktonic phase of this isolate. Exposure to RIF resulted in a limited reduction of 2-log CFU/mL for concentrations of ≥156.25 µg/L. Higher concentrations of CIP (≥ 25.6 mg/L) were required to reduce biofilm-residing bacteria by ≥2 log CFU/mL, with a maximum reduction of 2.8-log CFU/mL. Clindamycin exposure for 24 h maximally reduced the biofilm-residing bacteria by 0.6-log CFU/mL at the highest tested concentration (128 mg/L). None of the tested antibiotics were able to completely eradicate the biofilm-residing bacteria.

### Strategy 2: prolonged antibiotic exposure

**Fig 3:**
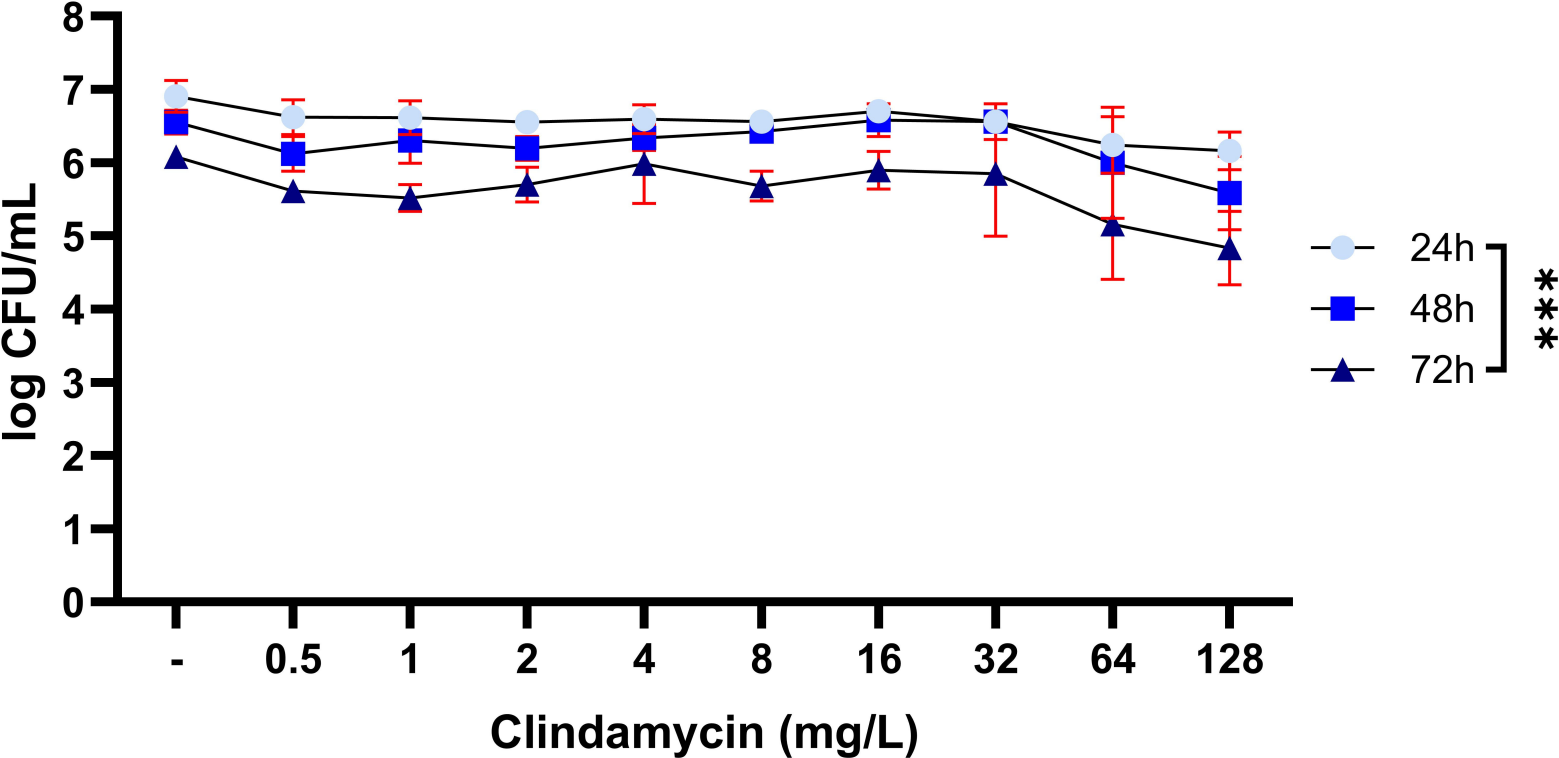
Bacterial load (CFU/mL) of mature 7-day LUH15392 biofilms, after exposure to various concentrations of clindamycin (mg/L) for 24, 48, or 72 h. Results are shown as log transformed mean values (*N* = 3 independent experiments, each performed in duplicate), with error bars indicating standard deviation. Two-way ANOVA was used to examine statistical differences between groups. ***P < 0.001. CFU: colony-forming units.

Next, we assessed the effect of prolonged exposure of mature biofilms to a single dose of clindamycin for up to 72 h (Fig. 3). Results showed a similar dose-dependent reduction in bacterial counts for 24-, 48-, and 72-h exposure to clindamycin, with a maximal reduction of 1.1-log CFU/mL at the highest tested concentration (128 mg/L).

### Strategy 3: repeated antibiotic exposure

**Fig 4:**
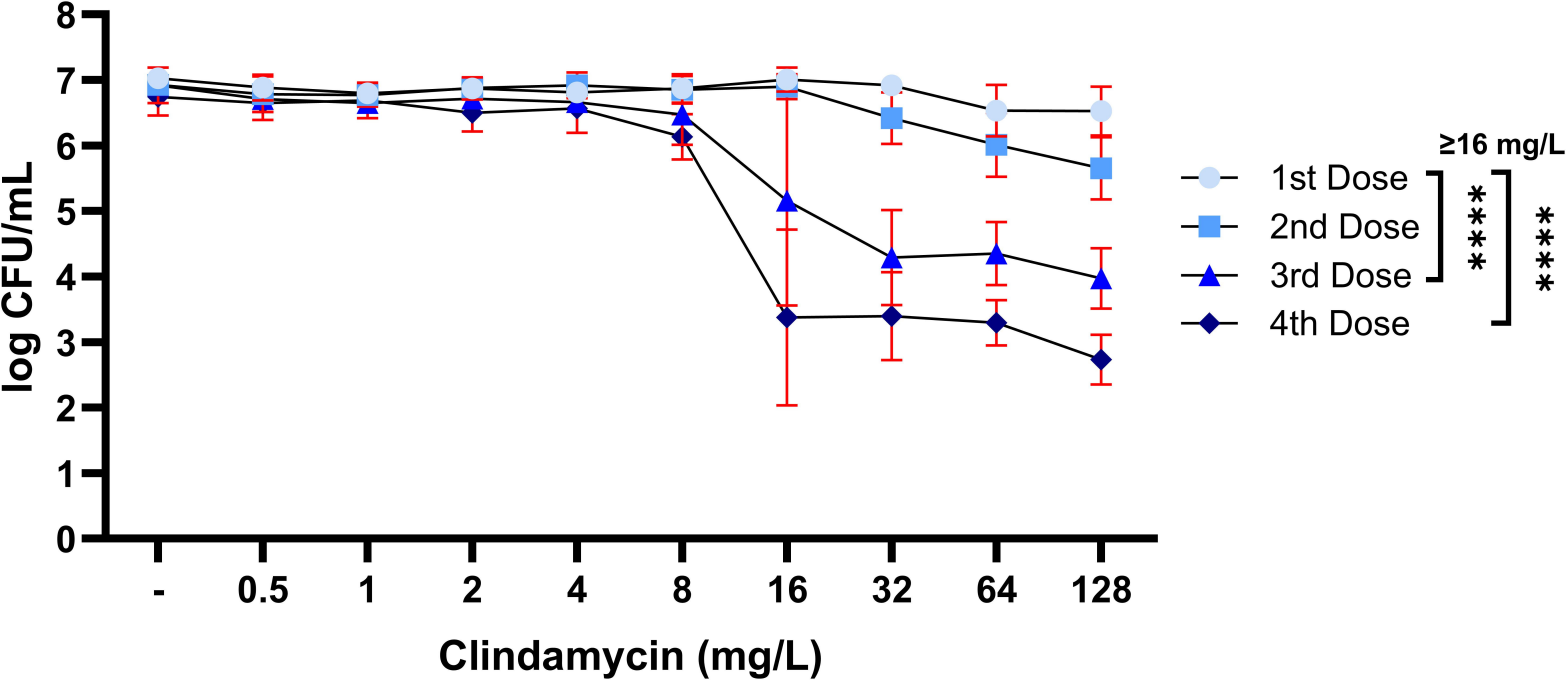
Bacterial load (CFU/mL) of mature 7-day LUH15392 biofilms, after exposure to various concentrations of clindamycin (mg/L) for one-, two-, three-, or four-time 24 h. Results are shown as log transformed mean values (*N* = 3 individual experiments, each performed in duplicate), with error bars indicating standard deviation. Two-way ANOVA was used to examine statistical differences between groups. ****P < 0.0001. CFU: colony-forming units.

Subsequently, we assessed the effect of repeated exposure to clindamycin (Fig. 4; Fig. S3). This approach better resembles the clinical treatment approach with multiple daily dosages (q8h), with our model using 24-h intervals. Results demonstrated that application of the first and second clindamycin dose did not effectively reduce *S. aureus* within mature biofilms. For the third dose, exposure to ≥8-mg/L clindamycin resulted in a decrease in bacterial load, with reductions up to 3.3-log CFU/mL. The fourth dose resulted in a further decrease in bacterial load for all concentrations ≥8 mg/L, with ≥16 mg/L reaching a reduction of 3-log CFU/mL. Overall, these results demonstrated that repeated exposure to clindamycin can be effective in reducing the amount of mature biofilm-residing bacteria with ≥3 log CFU/mL. However, complete eradication was not reached with this approach.

### Strategy 4: sequential antibiotic exposure

**Fig 5:**
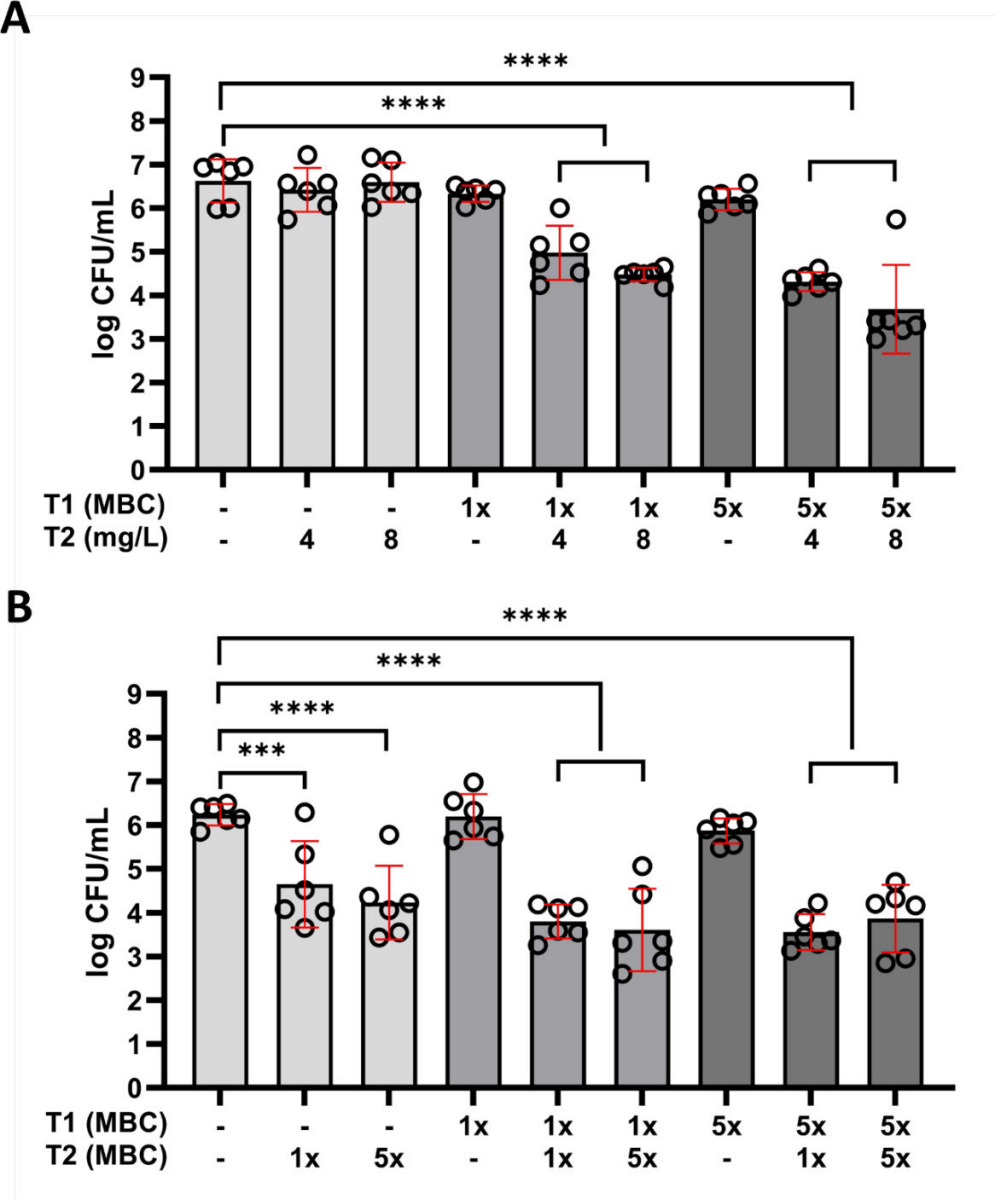
Bacterial load (log CFU/mL) of mature 7-day biofilm-residing *S. aureus* LUH15392 cultured on TAV discs, after 24-h exposure to RIF/CIP (T1), followed by (A) 24-h exposure to clindamycin (mg/L) (T2); (B) another 24-h exposure to RIF/CIP (T2). Results are shown as individual values (*N* = 3 experiments, performed in duplicate); bars indicate means, and error bars indicate standard deviation. One-way ANOVA was used to examine statistical differences between the control and antibiotic concentrations. ***P < 0.001; ****P < 0.0001. CFU: colony-forming units; MBC: minimum bactericidal concentration.

To further investigate potential clinical approaches for PJI treatment, a series of combinations were tested. First, the mature *S. aureus* biofilms were exposed to a combination of RIF and CIP in various concentrations for 24 h. This was followed by either single-dose clindamycin exposure, mimicking the oral switch strategy, or another dose of RIF/CIP, mimicking the standard approach. To approach the clinical situation of a biofilm-associated implant infection more closely, we assessed the effectiveness of sequential treatment using biofilms formed on Ti-6Al-4V implant material discs. For the sequential treatment strategy where 24-h exposure to RIF/CIP was followed by 24-h exposure to clindamycin (Fig. 5A), we observed the highest reduction in bacterial load (2.9-log CFU/mL) for secondary treatment with 8 mg/L. This reduction was similar to the 2.7-log CFU/mL reduction observed with repeated RIF/CIP exposures (Fig. 5B). Biofilms on plastic showed similar trends but more reduction compared to controls. Ultimately, the remaining fractions of biofilm-residing bacteria (mean 3.5- to 4-log CFU/mL) were similar to those observed in experiments on plastic (Fig. S4).

Since sequential treatment with clindamycin showed reductions of bacterial counts down to approximately 4-log CFU/mL, we cultured similar levels of these bacteria in continuous presence of clindamycin to assess regrowth of bacteria from this lower starting inoculum (Fig. S1B). Results further confirmed the capacity of clindamycin to inhibit bacterial growth at concentrations that are achievable in patients (4 and 8 mg/L), albeit for a shorter period of time (5 and 7 h, respectively). Furthermore, with these bacterial levels, ≥16-mg/L clindamycin completely eradicated all bacteria within 72 h without change of medium or additional antibiotics.

### Imaging of *S. aureus* biofilms after various treatment strategies

#### Confocal laser scanning microscopy

**Fig 6:**
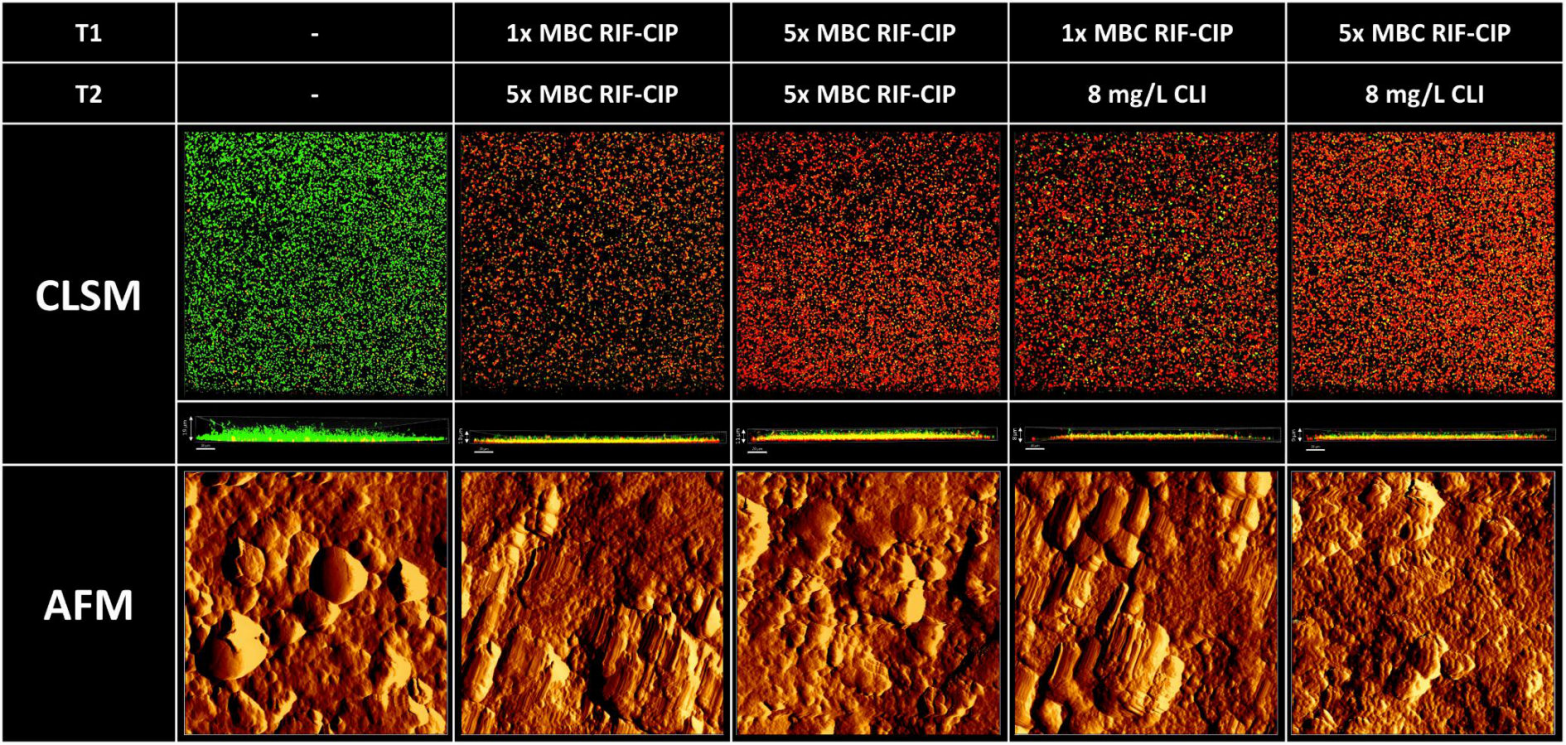
Visualization of mature 7-day biofilm-residing *S. aureus* LUH15392 on glass slides using confocal laser scanning microscopy (CLSM) (top) and on Ti-6Al-4V implant material discs using atomic force microscopy (AFM) (bottom). Images are divided by treatment: control biofilms are indicated by the - symbol; T1 indicates the first 24-h exposure, and T2 indicates the second 24-h exposure. Biofilm-embedded bacteria were visualized by LIVE (green)/DEAD(red) staining for CLSM. Apical biofilm is depicted on the bottom; basal biofilm is depicted on top of the z-stack. Scale bars indicate 20 µm. For the AFM, detailed 5 × 5 µm images were made at random locations on the biofilms. CIP: ciprofloxacin; CLI: clindamycin; MBC: minimum bactericidal concentration; RIF: rifampicin.

In addition to the microbiological determination of antibiotic effectivity, we imaged biofilms with confocal laser scanning microscopy (CLSM) to gain insight into the thickness of the biofilms and cell viability/death within (antibiotic-exposed) mature, 7-day biofilms (Fig. 6, top). CLSM of control biofilms showed a limited number of cells stained by the TO-PRO-3-iodide, while the various sequential exposure combinations resulted in a substantial increase in dead cells. This level of cell death corresponded with the remaining bacterial fractions observed after applying strategy 4, where the amount of bacteria (CFU/mL) was comparable between continued RIF/CIP and the alternative switch to clindamycin. Additionally, z-stack images depicted a decrease in the height of the biofilm after exposure (8–11 µm), compared to controls (19 µm). Visual analysis of the z-stacks demonstrated that cell death occurred throughout the biofilms, suggesting that there were no differences in the penetration of antibiotics between the different strategies.

#### Atomic force microscopy

Along with CLSM, we imaged the biofilms with AFM to gain insight into the biofilm structure and surface topography. Again, we compared untreated, mature biofilms to biofilms that were exposed to different antibiotics (Fig. 6, bottom). The untreated biofilms showed multilayered structures of bacterial cells and extracellular matrix, with visually smooth topography. The biofilms that were first treated for 24 h with RIF/CIP at 1× MBC showed elongated, rough bacterial cells and overall less smooth topography compared to controls. The biofilms with initial 24-h treatment with 5× MBC showed little to no identifiable bacterial cells and an overall disrupted topography. No clear differences were observed for the variations in second exposures to either RIF/CIP or clindamycin.

## DISCUSSION

In this study, we determined the *in vitro* antimicrobial activity of clindamycin as a monotherapy option against 7-day mature biofilm-residing *S. aureus*, compared to the standard RIF-based strategies. Additionally, we assessed different exposure strategies with clindamycin to determine the most effective approach.

We observed that initial exposure to clindamycin was—in contrast to planktonic bacteria—insufficiently effective in reducing biofilm-residing bacteria, especially compared to RIF-based strategies. Repeated dosing with clindamycin provided additional reductions but only at supraphysiological concentrations. In contrast, RIF-based exposure followed by clindamycin at physiological concentrations reduced biofilm-residing bacteria similar to strategies continuing with the RIF-based regimen. These results were also observed on a more clinically relevant implant material biofilm model, indicating that this approach can also be effective with biofilms on implants. These findings were further supported by analyses of imaging with CLSM and AFM, showing comparable effects on biofilm thickness, cell death within the biofilm, and disruption of the surface topography between continuation with RIF/CIP combination and the switch to clindamycin.

A limited number of clinical studies have previously evaluated the strategies related to our *in vitro* study. In a prospective, registry-based cohort of patients, clindamycin-based treatments in the context of PJIs were studied. The effects of these regimens were comparable to or even more effective than other therapeutic strategies (12). Our study was capable of reproducing these findings *in vitro* and therefore provides a valuable tool for further investigations into these observations. Earlier observational studies have indicated that steady-state concentrations in clindamycin monotherapy were more effective in combating PJIs than clindamycin–RIF combination therapy (17, 18). Combining clindamycin with RIF resulted in a 40% decrease in median clindamycin plasma concentrations, providing a strong indication to use clindamycin as a single agent. Notably, despite this significant decrease in clindamycin levels, this did not result in higher treatment failure rates. Additionally, serum concentrations were higher after intravenous administration, but oral administration provided better bioavailability and safety (19, 20). In clinical practice, the dosing of clindamycin already compensates for the significant loss of free antibiotic due to the variable but substantial protein binding of 60%–90% (21, 22). We based the concentrations used in our model on the free peak serum concentration (19, 23), indicating that clindamycin could be as effective as our data indicate. In our current *in vitro* model, it is difficult to closely mimic the clinical situation. To assess the potential loss of efficacy, we performed additional experiments based on strategy 4, the sequential treatment, where we added an approximation of albumin concentrations in humans (40 g/L). This did not result in any significant differences in antimicrobial efficacy (data not shown). These findings, together with our *in vitro* results, provide a strong argument for the switch to clindamycin monotherapy in second-phase PJI treatment.

The sequential exposure of 7-day biofilms to RIF/CIP for 24 h, followed by clindamycin for 24 h, is an approach that models an alternative clinical treatment strategy for PJIs. In the clinical approach, the second phase starts after 14 days of RIF-based treatment. In this second phase, clindamycin can be used as 600 mg q8h. Our model mimics the switch from RIF-based treatment to clindamycin monotherapy, with the main differences being the length and interval of dosing. However, the results showed considerable reductions in bacterial load, comparable to the continuation of a RIF-based regimen, even at a dosing interval of q24h. It is important to note that this model does not account for the breakdown and half-life of antibiotics in humans, or for the dynamic flow and distribution throughout the body. Our exposure strategies probably do not have the same progressive decrease in antibiotic concentrations over time or apply a continuous antibiotic infusion to maintain a constant level, therefore making results more difficult to extrapolate to the human situation.

For this study, we used a clinical *S. aureus* strain isolated from a PJI patient. We chose this isolate from a group of clinical PJI staphylococcal strains, which had similar susceptibility profiles regarding the antibiotics tested in this study. The growth kinetics and biofilm-forming capacities of these other PJI strains were assessed and were considered comparable for all selected *S. aureus* strains, with bacterial load (CFU/mL) of biofilm-residing bacteria remaining stable over a 7-day period (data not shown). More clinical strains, both MSSA and methicillin-resistant S. aureus (MRSA), could be tested to get a better understanding of the diversity of clindamycin susceptibility in PJIs. This could even be expanded to coagulase-negative staphylococci, for example, *Staphylococcus epidermidis*, which also includes this major group of causative pathogens of PJIs. Of note, MBC values were determined according to EUCAST (24) with a starting inoculum of 5 × 10^5^ CFU/mL (data not shown). This showed that the same concentrations were needed in this setup compared to our killing assay, described in Section 2.3. For uniformity of the model and comparison to the mature biofilms, we showed the results for the higher starting inoculum of 1 × 10^7^ CFU/mL.

The mature, 7-day biofilm model is based on the following considerations: first, in most clinical biofilm-associated infections like PJI, a mature biofilm has developed. Patients often develop clinical symptoms of PJIs within days, weeks, or even months after initial surgery, depending on the time of onset (25). In this period, the pathogen has had time to colonize the implant and develop a mature biofilm. Secondly, the heterogeneous biofilm structure with extracellular polymeric substances, DNA, and proteins makes drug penetration more difficult. Thirdly, previous work from our group (15) demonstrated a considerable difference in the phenotype of biofilms when assessed with AFM. An immature, 24-h model showed clustering of individual bacterial cells, with the implant material substrate still visible underneath. The mature, 7-day biofilm model demonstrated more pronounced biofilm structuring with the implant material substrate completely covered by layers of bacterial cells. The bacterial cells themselves were partially surrounded or fully covered by extracellular matrix. Further, clinically relevant strategies could be investigated with this model such as levofloxacin or non-fluoroquinolone-containing regimens like tetracyclines.

Despite reductions of biofilm-residing *S. aureus* below the 99.9% eradication threshold after antibiotic therapy, complete eradication was a rare occurrence in our model. The remaining bacterial fractions commonly consisted of approximately 3- to 4-log CFU/mL, which we also observed in our previous work on MRSA biofilms, with different treatments (15). These remaining fractions may expand rapidly and cause a relapse of disease in a clinical situation (26). We have observed that clindamycin is capable of inhibiting the growth of such bacterial concentrations in the planktonic phase. We can speculate that prevention of outgrowth of the fraction of surviving bacteria within the biofilm may also be due to the protective role of the hosts immunity. However, the clinical experience of relapsing infections after quitting long-term antibiotic treatment makes this hypothesis less likely. The addition of site-specific immune cells to the mature biofilm model could provide more insight into the role of the host immunity in eradicating these biofilms. For example, macrophages will respond to infection but may have difficulties phagocytosing the large biofilm structures. Antimicrobial therapies may be of aid here, by disrupting the biofilm structure and clearing a path for immune cells to have an effect. Combining these aspects into more advanced models could bridge the existing gap between simple *in vitro* and limited numbers of *in vivo* models, to gain more insight in the complex host–pathogen interaction that occurs during biofilm-associated implant infections.

To summarize, we assessed the effectiveness of clindamycin as a monotherapy for biofilm-associated PJIs in different *in vitro* scenarios. Our results showed that clindamycin as a single agent, at clinically relevant concentrations, was not effective in reducing the number of biofilm-associated bacteria. However, administration of these concentrations in second-phase treatment was as effective as continuation with RIF-based treatment. This strategy may provide an alternative approach, in which second-phase treatment can be switched to a single agent with generally less side effects, increasing the biological plausibility for patients.

## SUPPLEMENTAL MATERIAL

Supplemental material spectrum.01947-24-s0001.docx Table S1; Fig. S1 to S4.

ASM does not own the copyrights to Supplemental Material that may be linked to, or accessed through, an article. The authors have granted ASM a non-exclusive, world-wide license to publish the Supplemental Material files. Please contact the corresponding author directly for reuse.
