# Supplementary material for: Effectiveness of clindamycin-based exposure strategies in experimental mature staphylococcal biofilms
Source: Microbiol Spectr. 2025 Jan 30;13(3):e01947-24. doi: 10.1128/spectrum.01947-24 (PMC11878082; doi:10.1128/spectrum.01947-24)
Supplement: Supplemental material — Table S1; Fig. S1 to S4. [file spectrum.01947-24-s0001.docx]

**Appendices**

1. **Detailed step-by-step procedure for the different treatment strategies**

Table S-1: Step-by-step experimental setup of the different treatment strategies, with numbers corresponding to the order in which each step is executed.

| **Strategy 1** | **Strategy 2** | **Strategy 3** | **Strategy 4** |
| --- | --- | --- | --- |
| 1. Incubate 7 days at 37 °C 2. Wash biofilm 2x with 100 µL PBS 3. Add 100 µL of treatment per well 4. Incubate 24 hours at 37 °C 5. Wash biofilm 2x with 100 µL PBS 6. Sonicate 10 min. at 40 kHz to detach biofilms from wells 7. Dilute and plate well contents 8. Incubate plates at 37 °C | 1. Incubate 7 days at 37 °C 2. Wash biofilm 2x with 100 µL PBS 3. Add 100 µL of treatment per well 4. Incubate 48- / 72 hours at 37 °C 5. Wash biofilm 2x with 100 µL PBS 6. Sonicate 10 min. at 40 kHz to detach biofilms from wells 7. Dilute and plate well contents 8. Incubate plates at 37 °C | 1. Incubate 7 days at 37 °C 2. Wash biofilm 2x with 100 µL PBS 3. Add 100 µL of treatment per well 4. Incubate 24 hours at 37 °C 5. Wash biofilm 2x with 100 µL PBS 6. Repeat steps 4 and 5 for each dose 7. Sonicate 10 min. at 40 kHz to detach biofilms from wells 8. Dilute and plate well contents 9. Incubate plates at 37 °C | 1. Incubate 7 days at 37 °C 2. Wash biofilm 2x with 100 µL PBS 3. Add 100 µL of treatment per well 4. Incubate 24 hours at 37 °C 5. Wash biofilm 2x with 100 µL PBS 6. Add 100 µL of second phase treatment per well 7. Incubate 24 hours at 37 °C 8. Sonicate 10 min. at 40 kHz to detach biofilms from wells 9. Dilute and plate well contents 10. Incubate plates at 37 °C |


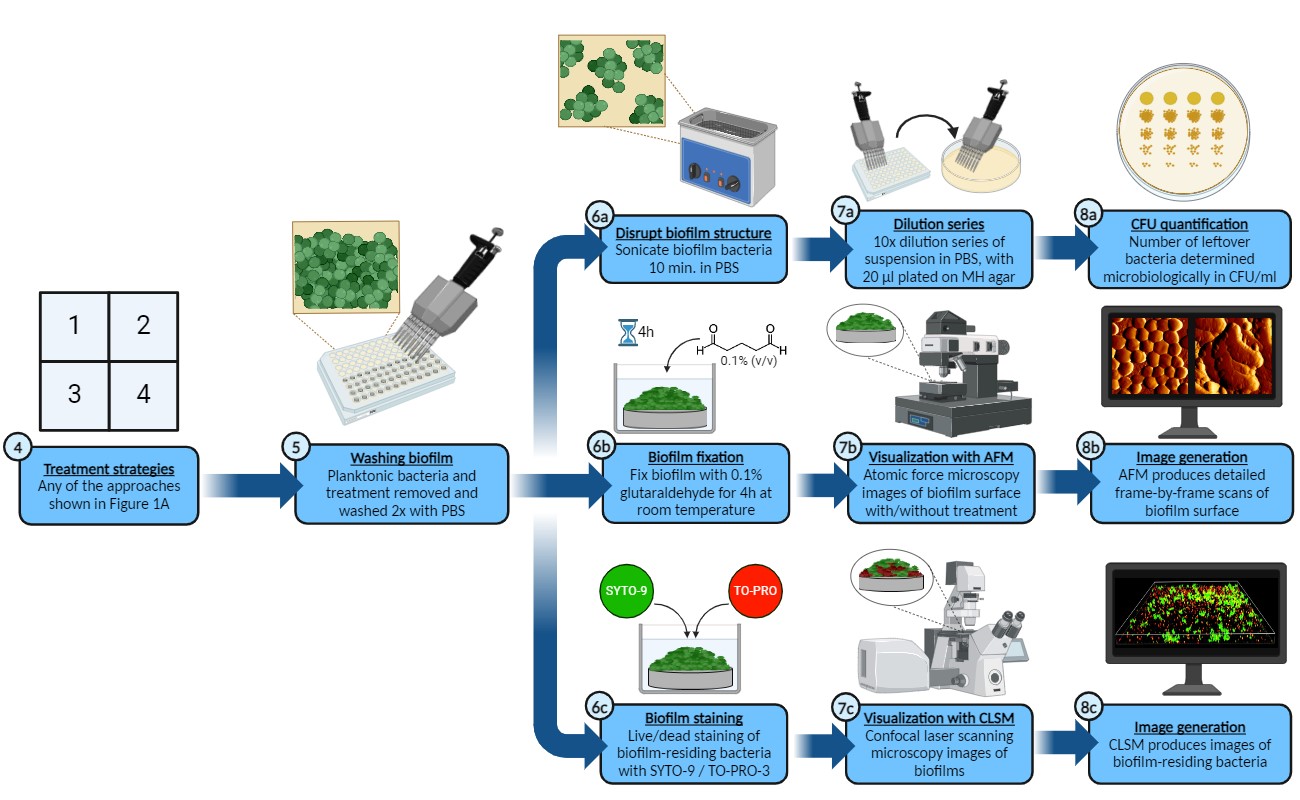


Figure S-1: Experimental approach of the different readouts of the anti-biofilm assays, with each number indicating a step in the procedure. The different readouts used in this study are indicated by steps 6 to 8. AFM: Atomic force microscopy; CFU: Colony forming units; CLSM: confocal laser scanning microscopy; MH: Mueller-Hinton; PBS: Phosphate buffered saline; TAV: Ti-6Al-4V implant material discs (Created with Biorender.com)

1. **Bacterial load of planktonic *S. aureus* under continued exposure to clindamycin**


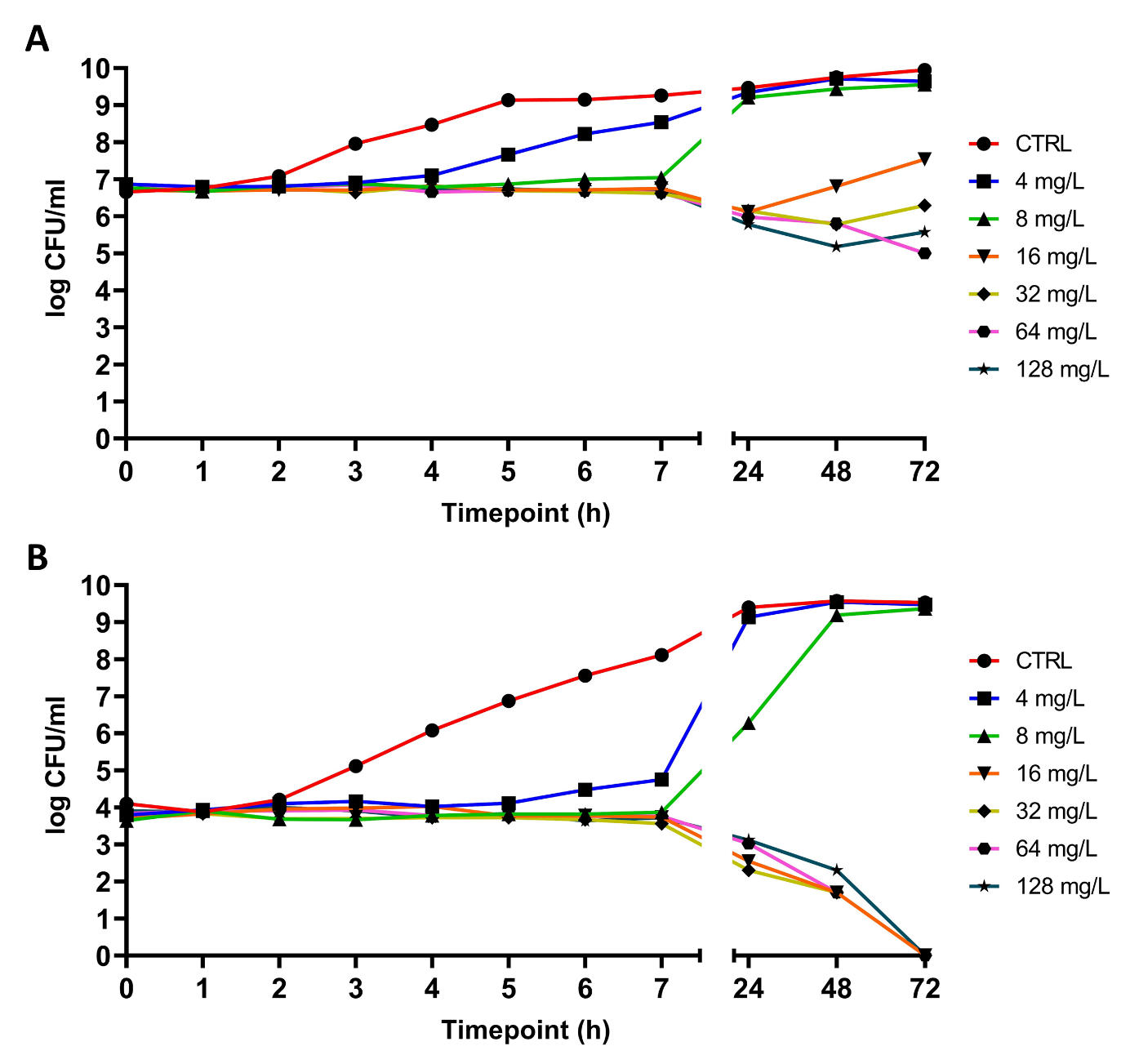


Figure S-2: Bacterial load (log CFU/mL) of planktonic S. aureus LUH15392 over time (h), incubated with various concentrations of clindamycin (mg/L) up to 72 h with a) starting at 10^7^ CFU/mL; b) starting at 10^4^ CFU/mL, mimicking the commonly leftover bacteria after biofilm treatment. Figure legend indicates the various concentrations, ranging from 4-128 mg/L, with a negative control (CTRL). Results are depicted as individual values (N=1). CFU: Colony forming units

1. **Bacterial load of 7-day, mature *S. aureus* biofilm after repeated exposure to clindamycin, per dose**


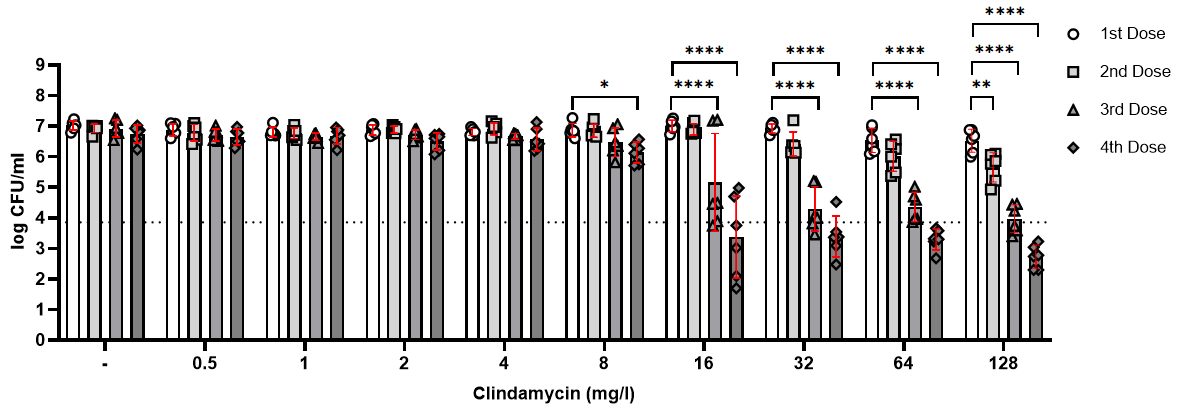


Figure S-3: Bacterial load (CFU/mL) of mature 7-day LUH15392 biofilms, after repeated exposure to various concentrations of clindamycin (mg/L) for 24 h. The different symbols and colors represent 1, 2, 3 and 4 times exposure to clindamycin for 24h, respectively. Results are shown as log transformed mean values (N=3 independent experiments, each performed in duplicate), with error bars indicating standard deviation. The dotted line represents the 99.9% eradication threshold. Two-way ANOVA was used to examine statistical differences between groups. * P < 0.05; ** P < 0.01; **** P < 0.0001. CFU: Colony forming units

1. **Bacterial load of 7-day, mature *S. aureus* biofilm after sequential exposure to rifampicin-ciprofloxacin combination followed by clindamycin**


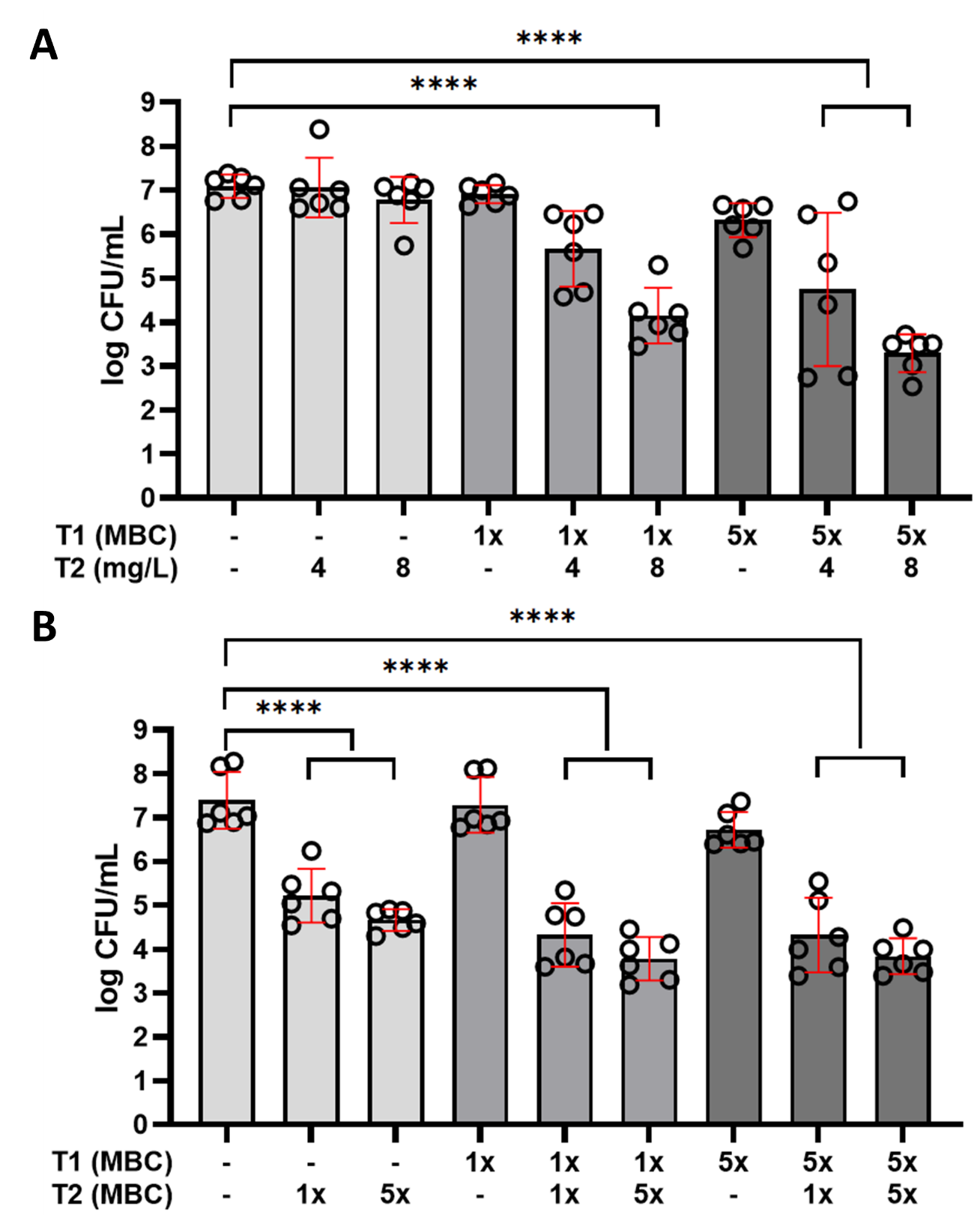


Figure S-4: Bacterial load (log CFU/mL) of mature 7-day biofilm-residing S. aureus LUH15392, after 24 h exposure to RIF/CIP (T1), followed by: a) 24 h exposure to clindamycin (mg/L) (T2); b) another 24 h exposure to RIF/CIP (T2). Results are shown as individual values (N=3 experiments, performed in duplicate), bars indicate means and error bars indicate standard deviation. One-way ANOVA was used to examine statistical differences between the control and antibiotic concentrations. *** P < 0.001; **** P < 0.0001. CFU: Colony forming units; MBC: Minimum bactericidal concentration.
